# Warming and Resource Availability Shift Food Web Structure and Metabolism

**DOI:** 10.1371/journal.pbio.1000178

**Published:** 2009-08-25

**Authors:** Mary I. O'Connor, Michael F. Piehler, Dina M. Leech, Andrea Anton, John F. Bruno

**Affiliations:** 1 Curriculum in Ecology, The University of North Carolina, Chapel Hill, Chapel Hill, North Carolina, United States of America; 2 Department of Marine Sciences, The University of North Carolina, Chapel Hill, Chapel Hill, North Carolina, United States of America; 3 Institute of Marine Sciences, Morehead City, North Carolina, United States of America; McGill University, Canada

## Abstract

Climate change disrupts ecological systems in many ways. Many documented responses depend on species' life histories, contributing to the view that climate change effects are important but difficult to characterize generally. However, systematic variation in metabolic effects of temperature across trophic levels suggests that warming may lead to predictable shifts in food web structure and productivity. We experimentally tested the effects of warming on food web structure and productivity under two resource supply scenarios. Consistent with predictions based on universal metabolic responses to temperature, we found that warming strengthened consumer control of primary production when resources were augmented. Warming shifted food web structure and reduced total biomass despite increases in primary productivity in a marine food web. In contrast, at lower resource levels, food web production was constrained at all temperatures. These results demonstrate that small temperature changes could dramatically shift food web dynamics and provide a general, species-independent mechanism for ecological response to environmental temperature change.

## Abstract 2

Experimental warming of a marine food web suggests that ocean warming can lead to greater consumer abundance but reduced overall biomass, providing a potentially species-independent response to environmental warming.

## Author Summary

Humans rely on marine ecosystems for economic and nutritional sustenance—including about 16% of animal protein consumed by humans—making it especially important for natural scientists, economists, conservationists and long-term policy planners to understand how climate change is likely to affect oceanic food webs. Yet the general effects of warming on food web productivity are completely unknown. The productivity of consumers (such as zooplankton), in food webs is determined in large part by their metabolic rates and the availability and productivity of their limiting metabolic resources. A general theory relating food web dynamics to temperature suggests that fundamental differences between consumers and primary producers (such as phytoplankton) may lead to predictable shifts in their relative abundance and productivity with warming. We experimentally tested the effects of warming on food web structure and productivity under two resource supply scenarios. Our results show that warming alone can strengthen the role of consumers in the food web, increasing consumer biomass relative to producer biomass, and reducing the total biomass of the food web despite increases in primary productivity. In contrast, when resources were less available, food web production was constrained at all temperatures. These results demonstrate that small changes in water temperature could drive dramatic shifts in marine food web structure and productivity, and potentially provide a general, species-independent mechanism of ecological response to climate change.

## Introduction

The ocean is a dynamic part of the global climate system. The temperature of the sea surface, where almost 50% of the world's primary productivity occurs [1], varies regionally as the result of changing surface air temperatures, currents, and upwelling of deeper water. Though links between climate conditions and pelagic food web productivity and structure have long been of interest to scientists [2], effects of physical conditions on secondary and tertiary productivity (hereafter: consumer productivity) have seemed too context dependent to allow general predictions [3]–[5].

**Figure 1:**
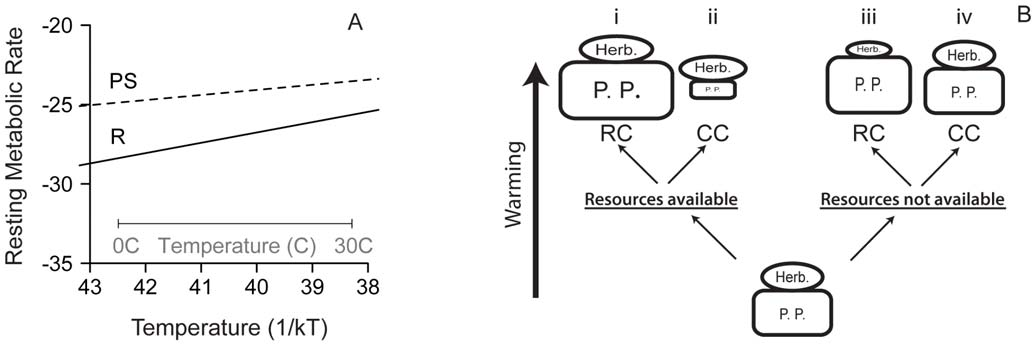
Effects of temperature on metabolism and food web structure. (A) Temperature (1/kT for T in Kelvin) dependence of photosynthesis- (PS, slope = −0.32 eV) and respiration- (R, slope = −0.65 eV) based mass-normalized resting metabolic rate (mmol O_2_/d pg C^αh^) (Adapted from Allen et al., 2005 [10], Lopez-Urrutia et al., 2006 [9]). (B) Four possible effects of warming on food web structure and biomass depend on resource availability and the importance of consumer-controlled (CC) or resource-controlled (RC) food web dynamics. Relative size of boxes and ovals indicate standing biomass stocks for the simplest of food webs comprising herbivores (Herb.) and primary producers (P. P.).

The prevailing conceptual framework for understanding effects of ocean temperature on food webs is based on the view that consumer production is predominantly controlled indirectly by temperature effects on primary production [6],[7]. According to this model, increased primary productivity and net autotrophy also increase CO_2_ uptake of the whole food web [8],[9]. Yet recently developed metabolic theory and a meta-analysis indicate that heterotrophic (respiration-limited) metabolism is more sensitive to changing temperature than autotrophic (photosynthesis-limited) metabolism and production (Figure 1A) [9],[10], suggesting stronger consumer-driven control with warming. Greater consumer control of primary production would lead to increased heterotrophy and less phytoplankton standing stock (Figure 1B-ii). In either model, the response of food web productivity and structure to changing environmental temperature may be determined by general processes and not the specific responses of component species, and thus could represent a critical step forward in efforts to forecast the impacts of climate change on ecological communities [11],[12].

Temperature-driven shifts in food web productivity and structure are limited ultimately by resource availability, and therefore must be considered in realistic nutrient supply contexts (Figure 1B) [5]. In the ocean, the same physical processes that drive temperature patterns also influence resource availability. Temperature-driven stratification isolates surface waters from cool, nutrient-rich deeper water, and because biological productivity at the sunlit surface depletes available nutrients, temperature and nutrient supplies are usually negatively correlated [5]. Nutrient limitation directly constrains primary production, while metabolic responses to temperature influence both photosynthetic and respiratory processes, and thus primary and consumer production. The metabolic effects of temperature therefore should be different and complementary to constraints imposed by resource availability.

**Figure 2:**
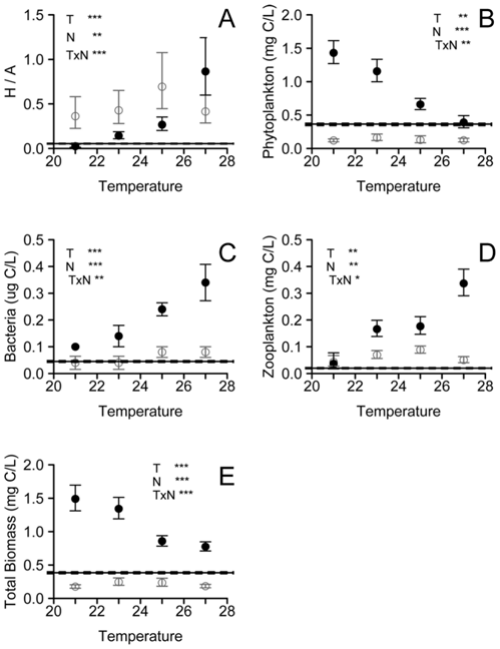
Effects of temperature and nutrient treatments on food web structure and biomass. Effect of temperature (°C) on mean (±s.e.) (A) ratio of heterotroph to autotroph biomass and the carbon biomass of (B) phytoplankton, (C) microbes, (D) zooplankton, and (E) the entire food web in nutrient addition (•) and control (○) treatments. Initial conditions (mean±s.e. indicated by horizontal lines) mimicked contemporary conditions in Bogue Sound. Significance of two-way ANOVA test: *** *p*<0.001, ** *p*<0.01, * p<0.05. Full statistical results in Table 2.

To understand the combined effects of temperature and resource availability on food web biomass (gC L^−1^) and productivity (gC L^−1^ yr^−1^), whole food web responses to variation in both factors need to be assessed. Using a coastal pelagic food web of phytoplankton producers and bacterial and zooplankton (>63 µm) consumers, we experimentally tested the effects of non-lethal temperatures and resource availability on food web structure (biomass allocation among trophic levels) and biomass standing stock (gC L^−1^). We assembled food webs in outdoor microcosms in a factorial experiment with four temperature levels (ambient, +2, +4, and +6°C) and two resource levels (nutrient additions and controls) (Table 1). Treatment levels mimic local estuary conditions during springtime warming and dry versus storm events causing riverine inputs of elevated nutrient concentrations (Figure S1). We measured effects of temperature and nutrient treatments on standing stocks of primary and secondary producers and on rates of primary productivity. Initial microcosm conditions included known amounts of zooplankton, phytoplankton, and bacteria (Figure 2) collected from the Bogue Sound Estuary at the University of North Carolina's Institute of Marine Sciences (IMS) in Morehead City, North Carolina.

**Table 1:** Experimental temperature and nutrient treatments.

	Temperature Treatment			
	Ambient	+2°C	+4°C	+6°C
Temperature °C *	20.4 (0.13)	22.7 (0.12)	24.0 (0.37)	26.4 (0.52)
Salinity *	34.7 (0.82)	36.4 (1.51)	36.4 (1.65)	38.2 (1.81)
**Nutrient Additions**	(initial µM)			
[NO_x_] (0.16)	0.03 (0.03)	0.00 (0.00)	0.10 (0.08)	0.07 (0.03)
[NH_4_] (0.55)	0.84 (0.20)	1.23 (0.38)	0.48 (0.07)	0.82 (0.11)
[PO_3_] ^†^ (0.05)	7.23 (0.38)	6.49 (0.50)	7.05 (0.68)	8.73 (0.50)
[TN] ^†^ (9.86)	30.98 (3.25)	28.04 (3.31)	31.16 (1.65)	32.86 (3.27)
**Nutrient Controls**				
[NO_x_]	0.03 (0.02)	0.03 (0.02)	0.03 (0.03)	0.05 (0.05)
[NH_4_]	0.97 (0.18)	0.65 (0.21)	0.59 (0.11)	1.07 (0.56)
[PO_3_] ^†^	0.02 (0.01)	0.04 (0.01)	0.09 (0.04)	0.03 (0.01)
[TN] ^†^	16.22 (3.32)	24.30 (5.03)	17.59 (1.28)	14.61 (3.50)

Mean (±s.e.) temperature based on hourly datalogger readings throughout the experiment. Temperatures fluctuated ±3°C daily similar to field conditions. Mean (±s.e.) final salinity and nutrient concentration values are given. Treatments received no nutrients (controls) or 20 µM N and 5 µM P on Days 0, 2, and 4 (additions). Significant (*p*<0.01, one- or two-way ANOVA) main effects of temperature* and nutrient^†^ treatments are indicated.

## Results

**Figure 3:**
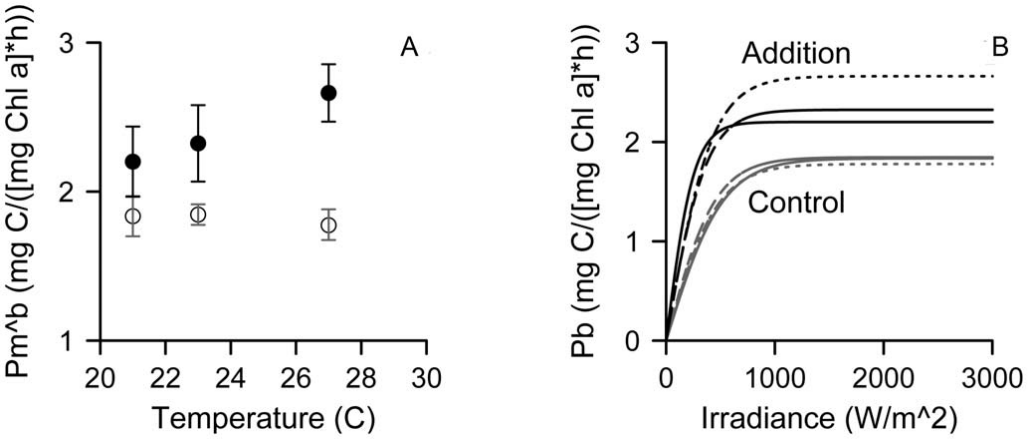
Effects of temperature on primary productivity. (A) Mean (±95% CI) maximum photosynthesis per unit chlorophyll biomass (P_M_^B^) in nutrient addition (•) and no-addition control (○) treatments and (B) P-I curves for nutrient addition (black lines) and no-addition control (gray lines) for ambient, +2, and +6°C treatments (solid, dashed, and dotted lines, respectively).

We found that small increases in temperature (Table 1) shifted food web structure toward greater heterotroph biomass relative to autotroph biomass (H/A) (Figure 2A). This shift is consistent with predictions based on differential temperature scaling of respiration- and photosynthesis-limited metabolism (Figure 1) [9],[13],[14]. Differential temperature scaling implies that organismal processes such as resource use, growth, and reproduction rates scale differently with temperature for heterotrophs and autotrophs [9],[14]. Consequently, increased grazing pressure with temperature dramatically reduced standing phytoplankton biomass in spite of increased per capita primary productivity (as approximated by the maximum photosynthesis per unit chlorophyll biomass, P_M_^B^, Figure 3, Table S1). Stronger consumer effects and greater consumer biomass were driven by higher density, and not increased individual size or a shift in the relative abundance of species (Figure S2). This pattern is consistent with the hypothesis that temperature affected change on a metabolic, individual level rather than via competitive exclusion or other species interactions.

Shifts in food web structure with warming were accompanied by a decrease in overall biomass (Figure 2E, Table 2). The decline in total biomass is consistent with stronger consumer control of food web structure with warming [14],[15], reflecting a direct effect of temperature on consumers and a disproportionate increase in grazing relative to primary production. Conversion of phytoplankton into consumer biomass is inefficient (∼10% [16]), so as consumers represent a greater proportion of the food web biomass, total biomass must decline. If instead the predominant influence of temperature on consumer productivity had been mediated indirectly by increased primary productivity, total food web biomass would have increased (Figure 2B-i). Although food web structures with reduced relative primary producer biomass are thought to be unstable, Carpenter et al. (2001) [15] showed that such a top-heavy food web structure can be sustained over time in natural pelagic lake food webs (i.e., at least 5 years).

**Table 2:** Statistical results of two-way ANOVA analyses on the effects of temperature and nutrient levels on biomass standing stocks in an experimental food web.

Response	Source	*df*	MS	*F*	*p*
Phytoplankton C biomass	Temperature	1	2.684	10.504	**<0.01**
	Nutrients	1	32.796	128.406	**<0.001**
	Temperature*Nutrients	1	2.293	8.976	**<0.01**
	Error	36	0.255		
Microbe C biomass	Temperature	1	4.798×10^−7^	23.621	**<0.001**
	Nutrients	1	8.404×10^−6^	41.376	**<0.001**
	Temperature*Nutrients	1	2.175×10^−7^	10.710	**<0.01**
	Error	36	2.031×10^−8^		
Zooplankton C biomass	Temperature	1	6.926	11.056	**0.002**
	Nutrients	1	6.798	10.851	**0.002**
	Temperature*Nutrients	1	4.504	7.189	**0.011**
	Error	36	0.627		
Heterotroph/Autotroph C biomass	Temperature	1	18.402	21.285	**<0.001**
	Nutrients	1	9.908	11.460	**0.002**
	Temperature*Nutrients	1	13.416	15.517	**<0.001**
	Error	36	0.865		
Total C biomass (mg/L)	Temperature	1	0.938	19.568	**<0.001**
	Nutrients	1	8.380	174.861	**<0.001**
	Temperature*Nutrients	1	0.950	19.825	**<0.001**
	Error	36	0.048		
Zooplankton average length (µm)	Temperature	1	3	<0.001	0.984
	Nutrients	1	93,345	14.448	**<0.001**
	Temperature*Nutrients	1	992	0.154	0.697
	Error	36	6,461		
Calanoid density	Temperature	1	9,193.7	18.434	**<0.001**
	Nutrients	1	13,468.9	27.006	**<0.001**
	Temperature*Nutrients	1	3,960.5	7.941	**0.008**
	Error	36	498.7		
Cyclopoid density	Temperature	1	105.13	1.375	0.249
	Nutrients	1	1,155.63	15.120	**<0.001**
	Temperature*Nutrients	1	235.44	3.080	0.088
	Error	36	76.43		
Harpacticoid density	Temperature	1	12.50	2.059	0.160
	Nutrients	1	16.90	2.783	0.104
	Temperature*Nutrients	1	2.00	0.329	0.570
	Error	36	6.07		
Nauplii density	Temperature	1	58,277	12.518	0.001
	Nutrients	1	121,000	25.992	<0.001
	Temperature*Nutrients	1	56,919	12.227	0.001
	Error	36	4,655		

Significant (*p*<*0.05*) results indicated in bold.

There was a strong interaction between resource availability and temperature effects on food web structure and total biomass. Nutrient addition allowed food web structure (H/A) to increase with warming and led to greater total food web biomass that declined with warming (Figure 2). In contrast, in the nutrient control treatments, resource availability constrained primary productivity at all temperatures (Figure 3), limited total standing biomass, and reversed the temperature-induced increase in H/A at the highest temperature. Low H/A at the highest temperature probably reflects exhaustion of resources at the highest productivity rates. In sum, resource availability determined whether food web structure was more strongly influenced by resources or by consumers (Figure 1B). If this experimental system is representative of effects of environmental warming, the interaction between nutrient supply and temperature suggests that in nutrient-poor regions, food webs may be more resilient to warming because consumer production is limited by resource availability, while in nutrient-rich regions small amounts of warming may have dramatic effects on trophic structure, primary productivity, and standing biomass.

Food web experiments in microcosms are a necessary but imperfect approximation of natural conditions. Microcosm experiments allow manipulation of environmental factors that would be impossible in the field while allowing natural feeding interactions, behaviors, and population growth processes to occur. These advantages undoubtedly enhance our understanding of fine-scale biological dynamics in pelagic ecosystems. Nonetheless, small microcosms impose several limitations on the broad interpretation of their results. For example, evaporation at warmer temperatures increased salinity in our microcosms (Table 1). Reduced concentrations of dissolved oxygen and carbon dioxide are also associated with warmer temperatures and likely varied naturally in our microcosms. Though these factors can influence productivity, their effects are negative and small over the experimental temperature range relative to the strong positive effects of temperature [17]. In addition, it is possible that the importance of consumer control was amplified in our experimental microcosms. For example, small experimental systems with relatively homogenous environments can facilitate foraging and reduce refuges for resources. Nonetheless, top-down determination of food web structure and dynamics has been documented in large-scale aquatic ecosystems [15],[18] and may become more important in a warming environment.

## Discussion

Temperature is known to influence food web structure [13],[19],[20], and such findings have generally been attributed to differential effects of resource limitation across trophic levels, or the specific effects of temperature on consumers or producers [13],[19]. Our experiments demonstrate that temperature alone can shift food web structure and change total standing biomass. Furthermore, biogeographic trends towards net heterotrophy in warmer climates in open ocean pelagic food webs [9],[21] and patterns observed in spring bloom dynamics, rocky intertidal systems, grasslands, and forests [20],[22]–[25] are consistent with differential metabolic scaling across trophic levels, though this mechanism has been invoked and tested in just one of these cases [9]. By explicitly testing the hypothesis based on metabolic theory in the context of food web ecology, we have for the first time experimentally validated the prediction that universal temperature constraints on individual metabolism can lead to general responses at the community level [11],[26].

The interaction between effects of temperature and nutrient availability observed in these experiments deepens our understanding of food web responses to changing climate conditions. In pelagic marine ecosystems, projected increases in stratification imply that negative correlations between nutrient availability and temperature will intensify in many regions [5],[27]. This pattern occurs on very broad geographic scales (i.e., cold temperate or polar systems relative to tropical systems), on smaller scales within oceans and seas (i.e., the North Sea [19]), and over time within a single region [4]. According to theory supported by our experimental results, small increases in sea surface temperature should cause small declines or no change at all in primary productivity and standing stocks in nutrient-poor systems such as stratified areas with a shallow thermo- or pycnocline. Under such conditions, nutrient limitation would constrain consumer productivity and biomass stocks (Figure 1B-iii), and could even lead to reduced consumer biomass with warming due to increased respiratory costs that exceed available primary production. In contrast, when nutrients are plentiful, as in upwelling or well-mixed systems, warming should increase productivity leading to increased biomass production at higher trophic levels, shifted food web structure, and stronger consumer control of phytoplankton standing stock (Figure 1B-ii).

The importance of temperature scaling of food web structure for fisheries productivity and food webs in aquatic ecosystems depends on the contemporary food web structure. In nature, most food webs include consumers at trophic levels higher than the zooplankton used in our microcosm experiments. In more complex food webs, temperature-driven intensification of consumer control could strengthen a trophic cascade, causing increased phytoplankton biomass as a result of indirect effects of increased consumption by carnivores. Alternatively, if consumer biomass has been severely reduced due to overfishing, direct effects of differential temperature scaling across trophic levels may be difficult to detect and indirect effects of increased primary productivity may be most apparent (Figure 1B-i) [3].

Ocean warming or cooling influences marine ecosystems in a variety of ways. For example, together with associated changes in physical properties such as vertical stratification and ice cover, warming has shifted species composition and altered the timing of seasonal spawning and spring bloom events [5],[28],[29]. The ramifications of these changes can be severe for some species and mild for others, causing mismatch between interacting species [6],[20],[28]. Temperature scaling of food web properties, however, is a general response to temperature change that should occur regardless of species composition [9],[10]. This mechanistic response can be incorporated into predictions of ecological variation, thus providing one of the few general models for ecosystem change with geography or climate.

The conceptual framework outlined here reinforces predictions that effects of climate change on ecosystem processes will vary among regions [7],[30]. Future warming will likely increase secondary productivity and fish harvests in nutrient-rich regions, but may cause little change in more stratified, oligotrophic systems. These are not paradoxical responses, and the general effects of temperature in different nutrient contexts explain why different responses to warming can occur within the same ecosystem. Implications of temperature effects on food webs for the ocean's role in carbon cycling are unclear, due in part to the mosaic of nutrient-rich and nutrient-poor regions of the world's oceans, and to temperature-driven shifts in the threshold dividing net heterotrophy from net autotrophic (carbon sinks from carbon sources) [9]. Nonetheless, small degrees of warming may have predictable broad scale consequences for the productivity and structure of aquatic ecosystems.

## Methods

### Mesocosm System and Experimental Design

Food webs were maintained in 4-L translucent plastic microcosms (*n* = 5) in outdoor water tables at IMS from April 23 to May 1, 2008. Pilot experiments indicated that 8 days were sufficient to allow zooplankton population growth without exhausting water quality. We maintained temperature treatments in a blocked design with temperature blocked by water table (Table 1). Temperature treatments were significantly different (one-way ANOVA with temperature as a continuous variable: *df* = 1, *F* = 567.72, *p*<0.001), and water table did not alter the treatment effects (comparison of nested linear models using likelihood ratio tests indicated no improvement by including a water table term: *p*>0.952). Temperatures were monitored regularly using a hand thermometer and continuously using ibutton Thermochron dataloggers (Dallas semiconductor, Dallas, Texas, USA). Nutrient addition and control replicates were randomly arranged in water tables. Plexiglass and one layer of window screen were placed several inches above microcosms to block UV radiation, minimize evaporation, and reduce light levels to those similar to 0.5–1.0 m depth (approximately 900 µM photons/m^2^/s midday on a sunny day), while still allowing unhindered gaseous exchange with the atmosphere. Each microcosm received air through an air stone to maintain oxygen levels and water mixing.

### Sampling Food Web Structure, Biomass, and Primary Productivity

Phytoplankton biomass was estimated by quantifying chlorophyll *a* concentrations in 50 mL aliquots of each replicate. Nutrient (NH_4_, PO_4_, NO_X_ and total nitrogen (TN)) concentrations were quantified using the filtrate from the same water samples used to estimate phytoplankton biomass. Zooplankton were sorted from water remaining in the microcosm after other sampling (2,768 mL) using a 63 µm mesh and preserved in 4.5% sucrose Formalin. In the laboratory, zooplankton were counted and identified to lowest taxonomic level possible at 40× magnification. Carbon biomass was estimated by converting from chl a, ash free dry weight, and visual counts for phytoplankton, zooplankton, and microbes, respectively (Text S1).

Final maximum primary productivity was estimated using photosynthesis versus irradiance (P-I) relationships for ambient, +2, and +6°C. Maximum photosynthesis per unit chlorophyll biomass (P_M_^B^) and the initial slope of the P-I curve (±95% confidence intervals) were calculated based on estimation of radioactive carbon uptake at each treatment level. Phytoplankton samples were collected from microcosms and spiked with 14C-bicarbonate (Amersham) to a final concentration of 0.8 µCi mL^−1^ and incubated for 45 minutes at varied irradiances (Text S1).

Effects of temperature and nutrient levels on response variables were analyzed using a two-way ANOVA. Biomass data were log-transformed prior to analysis to meet the assumptions of ANOVA. All statistical analyses were performed in R (v. 2.7.0). P-I curve fitting was performed in SAS.

## Supporting Information

Figure S1 **Natural variation in nutrient concentrations (µM) and temperature (°C) in Bogue Sound, North Carolina.**
(3.60 MB RTF)

Figure S2 **Effect of temperature (°C) on zooplankton size, density, and taxonomic composition.**
(0.34 MB PDF)

Table S1 **Parameters for photosynthesis-irradiance (P-I) curves.**
(0.06 MB RTF)

Text S1 **Additional methodological detail.**
(0.05 MB RTF)

## Abbreviations

IMS: Institute of Marine Sciences
H/A: heterotroph biomass relative to autotroph biomass
TN: total nitrogen
P-I: photosynthesis versus irradiance
