# Supplementary material for: Warming and Resource Availability Shift Food Web Structure and Metabolism
Source: PLoS Biol. 2009 Aug 25;7(8):e1000178. doi: 10.1371/journal.pbio.1000178 (PMC2723928; doi:10.1371/journal.pbio.1000178)
Supplement: Figure S1 — Natural variation in nutrient concentrations (µM) and temperature (°C) in Bogue Sound, North Carolina. (3.60 MB RTF) [file pbio.1000178.s001.rtf]

Figure S1.  Natural variation in nutrient concentrations (µM) and temperature (C) in Bogue Sound, North Carolina.  Monthly means (+ s.e.) given for 2007-2008.


 
