# Supplementary material for: Warming and Resource Availability Shift Food Web Structure and Metabolism
Source: PLoS Biol. 2009 Aug 25;7(8):e1000178. doi: 10.1371/journal.pbio.1000178 (PMC2723928; doi:10.1371/journal.pbio.1000178)

Warming and resource availability shirt food web structure and metabolism.
O'Connor et al

Figure S2: Effect of temperature (°C) on zooplankton size, density and taxonomic composition. Mean ± se **A)** density and **B)** average length (μm), and **C)** average biomass (μg C), and densities of nauplii ▲, calanoids ●, cyclopoids ■ and harpacticoids ◆ in **D)** nutrient (filled symbols) and **E)** no nutrient (open symbols) treatments. Initial conditions (mean ± s.e.) indicated by horizontal lines.

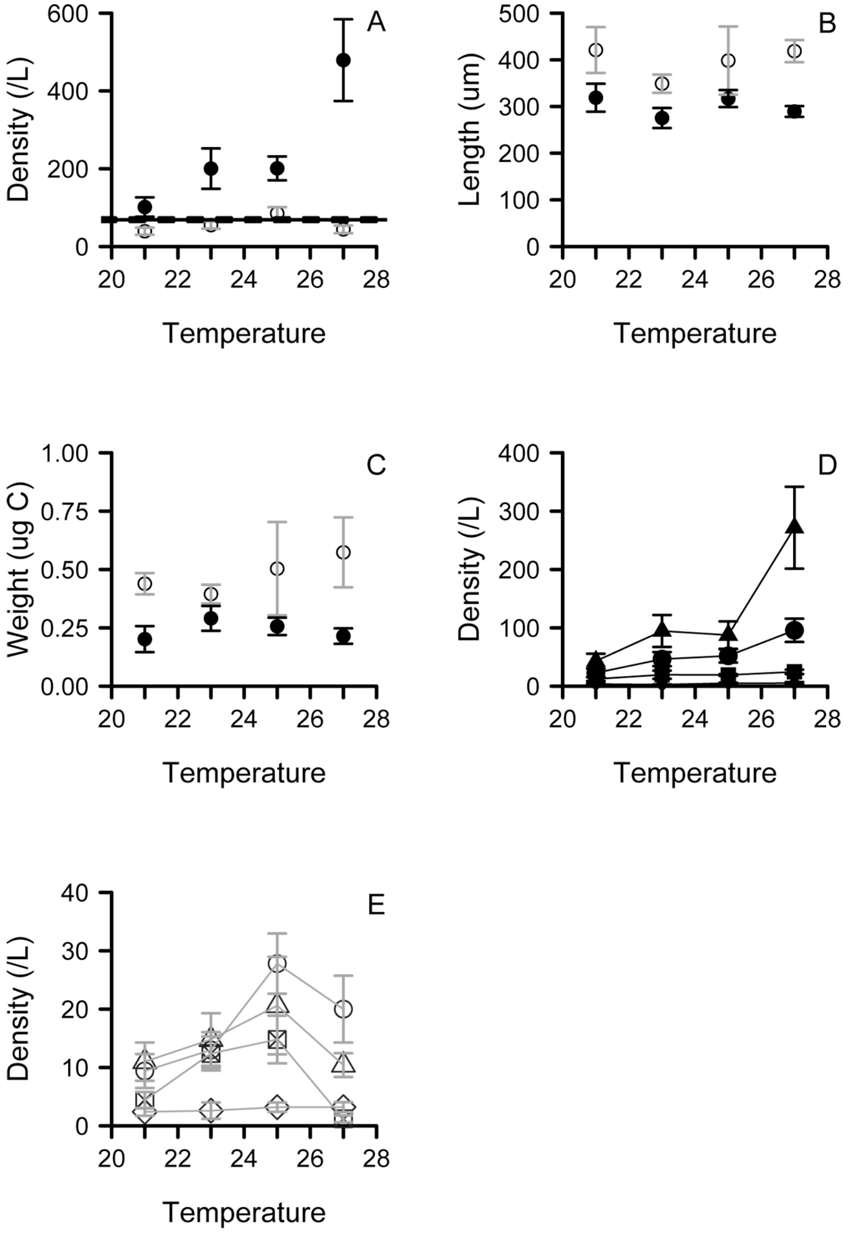

Supplement: Figure S2 — Effect of temperature (°C) on zooplankton size, density, and taxonomic composition. (0.34 MB PDF) [file pbio.1000178.s002.pdf]
