# Supplementary material for: Warming and Resource Availability Shift Food Web Structure and Metabolism
Source: PLoS Biol. 2009 Aug 25;7(8):e1000178. doi: 10.1371/journal.pbio.1000178 (PMC2723928; doi:10.1371/journal.pbio.1000178)
Supplement: Table S1 — Parameters for photosynthesis-irradiance (P-I) curves. (0.06 MB RTF) [file pbio.1000178.s003.rtf]

Table S1.  Parameters for photosynthesis-irradiance (P-I) curves (+ 95% confidence intervals): initial slope () and maximum photosynthesis per unit chlorophyll biomass (PMB) [5].
No nutrients	 (mg C *[mg Chl a]-1 hr-1 / W m-2)	PmB  (mg C/[mg Chl a]/hr)	
Ambient	0.00354 (0.00268-0.00439)	1.8354 (1.7004-1.9704)	
+2 C	0.00411 (0.00358-0.00464)	1.846 (1.777-1.915)	
+6 C	0.0038 (0.00305-0.00455)	1.7785 (1.6754-1.8816)	
			
Nutrient addition		
Ambient	0.00862 (0.00564-0.0116)	2.2013 (1.966-2.4367)	
+2 C	0.00632 (0.00458-0.00807)	2.3241 (2.0676-2.5806)	
+6 C	0.00629 (0.00522-0.00732)	2.6623 (2.4693-2.8553)	


 
