# Supplementary material for: Warming and Resource Availability Shift Food Web Structure and Metabolism
Source: PLoS Biol. 2009 Aug 25;7(8):e1000178. doi: 10.1371/journal.pbio.1000178 (PMC2723928; doi:10.1371/journal.pbio.1000178)
Supplement: Text S1 — Additional methodological detail. (0.05 MB RTF) [file pbio.1000178.s004.rtf]

Supplementary Text 1.

Warming and Resource Availability Shift Food Web Structure and Metabolism

Mary I. O'Connor1,3,†, Michael F. Piehler2, Dina M. Leech2, Andrea Anton1,3 and John F. Bruno3

1 Curriculum in Ecology, CB 3275, The University of North Carolina, Chapel Hill, NC, 27599
2 Institute of Marine Sciences, 3431 Arendell Street, Morehead City, NC 28557
3 Department of Marine Sciences, CB 3300, The University of North Carolina, Chapel Hill, NC, 27599

† Present address: National Center for Ecological Analysis and Synthesis, 735 State Street, Suite 300, Santa Barbara, CA 93101


Methods:
Experimental conditions and organism collection
Microcosms started with identical conditions of 3 L of filtered seawater containing phytoplankton and microbes at contemporary field densities (6.64 mg Chl a/L and 2.23 x 10^6^ / L, respectively) and an inoculum from the concentrated pool of zooplankton (69/L, consistent with measured field densities [1]).  We collected zooplankton, phytoplankton and microbes from the Bogue Sound Estuary at the University of North Carolina's Institute of Marine Sciences (IMS) in Morehead City, NC, on 4/23/07 in the mid-morning just before high tide.  Phytoplankton and microbes were separated from particulates and zooplankton using a 63-m filter, and zooplankton were collected using a 63-m net.  Zooplankton and the phytoplankton and microbes were stored in separate coolers for less than one hour prior to the start of the experiment.

Sampling food web structure, production and primary productivity
Phytoplankton was concentrated on Whatman GF/F glass fiber filters (25mm diameter, 0.7 m nominal pore size) and chlorophyll a was extracted using 10 mL acetone.  Pigment levels were determined using a Turner Designs Trilogy fluorometer using the non acidification module [2].  Carbon biomass was estimated using a conversion factor of 55 mg C per mg Chl a [3].  Nutrients were quantified with a Lachat Quick-Chem 8000 automated ion analyzer using standard protocols (Lachat Instruments, Milwaukee, WI, USA: NO2/NO3 Method 31-107-04-1-C, NH4 Method 31-107-06-1-A, PO4 Method 31-115-01-3-C, and TN Method 31-107-04-3-B).

Zooplankton body length was estimated for a subset of individuals counted, and biomass (ash free dry weight, AFDW) was determined for a subset of each replicate.  Carbon biomass was estimated by multiplying AFDW by 0.5 [4].  Bacteria were collected in 3 mL aliquots and preserved in glutaraldehyde.  Subsamples were stained with DAPI for 10 minutes before 1 mL of sample, combined with 1 mL of sterilized deionized water, was filtered onto 0.22 m black polycarbonate filters.  Filters were then mounted onto microscope slides and viewed under oil immersion on an epifluorescence microscope at 1000x magnification.  Approximately 400 cells were counted per slide to estimate microbial abundance using the equation

Bacteria/mL = (membrane conversion factor x N)/D

Where N is the average number of bacteria per field of view, the membrane conversion factor is the filtration area per field of view area, and D is the dilution factor.  Carbon biomass was estimated by multiplying counts by 20 fg C / bacterium.

Parameters for photosynthesis versus irradiance (P-I) relationships were calculated according to Jassby and Platt [5].  P-I relationships were determined using the Lewis and Smith (1983) [6] photosynthetron method.  Water samples (60 mL) were collected from each replicate microcosm and pooled for each temperature x nutrient treatment.  Samples of 5 mL from this pool were spiked with 14C-bicarbonate (Amersham, Inc.) to a final concentration of 0.8 µCi mL-1.  Samples were incubated for 45 minutes at varied irradiances generated by two Cool-Lux 75 W projector lamps using a combination of neutral density filters, distance from light source, and angle of incidence [6].  One mL of 50% HCl was added to the samples which were then placed on a shaker table overnight to purge unincorporated 14C.  Ten mL of Ecolume (ICN Inc.) scintillation cocktail was added to each vial, which were stored in the dark for 12 hours and were then counted in a Beckman model LS 5000TD liquid scintillation counter.  Counts per minute were converted to disintegrations per minute using internal quench curves from a calibrated 14C-toluene standard. 

References:

1.  Fulton RS, III (1984) Distribution and community structure of estuarine copepods.  Estuaries 7:38-50.
2.  Welschmeyer NA (1994) Flourometric analysis of chlorophyll a in the presence of chlorophyll b and phaeopigments. Limnol Oceanogr 39: 1985-1992.
3.  Gasol JM, del Giorgio PA, Duarte CM (1997) Biomass distribution in marine planktonic communities. Limnol Oceanogr 42: 1353-1363.
4.  Cebrian J (1999) Patterns in the fate of production in plant communities. Am Nat 14: 449-468.
5.  Jassby AD, Platt T (1976) Mathematical forumulation of relationship between photosynthesis and light for phytoplankton. Limnol Oceanogr 21: 540-547.
6.  Lewis MR, Smith JC (1983) A small volume, short-incubation-time method for measurement of photosynthesis as a function of incident irradiance. Mar Ecol Prog Ser 13:99-102.
